# Beware of subgroup analysis

**DOI:** 10.1007/s00467-008-0791-4

**Published:** 2008-07-01

**Authors:** Mark M. Mitsnefes, Philip R. Khoury, Prasad Devarajan

**Affiliations:** 1 Divisions of Nephrology and Hypertension, Cincinnati Children’s Hospital Medical Center, University of Cincinnati School of Medicine, Cincinnati, OH USA; 2 Division of Cardiology, Cincinnati Children’s Hospital Medical Center, University of Cincinnati School of Medicine, Cincinnati, OH USA; 3 Nephrology and Hypertension, MLC 7022, 3333 Burnet Avenue, Cincinnati, OH 45229–3039 USA

**Keywords:** Chronic Kidney Disease, Subgroup Analysis, Kidney Function, Receiver Operating Characteristic Analysis, Pediatric Nephrology

We appreciate the opportunity to respond to the thoughtful comments submitted by Dr. Lemaire [1] about our article titled “Serum neutrophil gelatinase-associated lipocalin as a marker of renal function in children with chronic kidney disease” in *Pediatric Nephrology* [2]. After careful reassessment of the data, we stand by the results and conclusions reported in our article.

Dr. Lemaire states that our work “is more suited for hypothesis generation.” Indeed, we concluded that “the present study *suggests* serum neutrophil gelatinase-associated lipocalin (NGAL) as a *candidate* for inclusion in a *panel* of biomarkers.” We stated important limitations of the study [cross-sectional design, small sample size, applicable only to young subjects with chronic kidney disease (CKD)], which also indicate the preliminary nature of the study and in no way makes this study conclusive.

Despite limitations of the statistical analysis, related primarily to the small sample size, the difference in relationship between NGAL, cystatin C, and estimated glomerular filtration rate (eGFR) at low levels of GFR persisted even after artificial manipulation of the data (i.e. removal of a data point for NGAL) performed by Dr. Lemaire. Using the actual data set, our analysis resulted in a decrease of *R* ^2^ value for GFR–NGAL from 0.38 to 0.26 and not to 0.20, as reported in Dr. Lemaire’s letter [1]. The *R* ^2^ of 0.26 still represents a significant improvement over the cystatin C–GFR (*R* ^2^ = 0.08) or eGFR–GFR (*R* ^2^ = 0.12) relationships. We also feel that there is no justification to arbitrarily consider one of the NGAL data points as an outlier, as suggested in Dr. Lemaire’s letter [1].

In Dr. Lemaire’s letter, the clustering of points in the graph of eGFR and GFR speaks only to these two variables, not to any other pair of variables. The fact that there is a negative correlation in this group, rather than being artifactual, may be due to the fact that eGFR does not work so well for a certain percentage of the patients in this subgroup analysis. A close examination of this “subgraph” shows a number of points fitting very nicely on the original regression line and a number of other points drifting out to the right of the line. We cannot discern whether there may be something different about these subjects compared with those that fit on the line so well. But why were there no points to the left of the regression line? Perhaps the GFR–eGFR relationship breaks down at this low level of GFR for some patients, depending on the underlying disease processes. In any case, we are not ready to concede that the negative correlation seen is artifactual in this case. We do concede that the correlations produced at a GFR <30 will provide limited information, as this only contains 20% of the range of GFRs in the study and therefore truncates this distribution. But this limitation was induced by using a widely accepted cutoff (GFR <30 ml/min per 1.73 m^2^). In any case, the correlation analyses, and specifically the subgroup analyses, were performed, as we stated in the manuscript, “to further investigate the relationships between studied biomarkers and measured GFR.” Our conclusions are not based on the subgroups analyses but on the results of the regression residual and receiver operating characteristics analyses. The subgroup analyses were presented only to confirm that the correlations seen in the whole group also held for subgroups, and there is no inconsistency at the low end of the GFR distribution.

The results, regardless of the *R* ^2^ value, still suggest that NGAL outperforms other markers of kidney function in our study. Does this statistical finding make NGAL a useful GFR surrogate? Definitely not, and in this respect, we agree with Dr. Lemaire. As we indicated in the article, larger confirmative studies are necessary to evaluate the role of NGAL and other potential biomarkers of kidney function in children with CKD. Further, as we suggested in the manuscript, the true value of NGAL may be confirmatory of progressive decline in kidney function, as it appears to perform especially well in the moderate range of reduced GFR.

Monitoring CKD activity requires biomarkers that provide clinicians with quick, noninvasive, and specific measurements that correlate with kidney pathophysiology. Current biomarkers of CKD and its progression, serum creatinine and urine protein, have serious limitations in serving these goals [3]. Remarkably, much of the controversy surrounding the early diagnosis of acute and chronic kidney disease is being solved by the adaptive response of the stressed kidney itself [4]. The application of innovative technologies has begun to identify novel biomarkers that reflect tissue pathology and predict disease progression prior to the development of abnormalities in traditional biomarkers [5]. NGAL holds promise as a method for monitoring CKD progression, and it has been gratifying to witness the recent literature providing further support to this notion [3].
